# An Economic Evaluation of Venous Thromboembolism Prophylaxis Strategies in Critically Ill Trauma Patients at Risk of Bleeding

**DOI:** 10.1371/journal.pmed.1000098

**Published:** 2009-06-23

**Authors:** T. Carter Chiasson, Braden J. Manns, Henry Thomas Stelfox

**Affiliations:** 1 Department of Biomedical Sciences, University of Calgary, Calgary, Canada; 2 Departments of Medicine and Community Health Sciences, University of Calgary, Calgary, Canada; 3 Departments of Critical Care Medicine, Medicine and Community Health Sciences, University of Calgary, Calgary, Canada; University College London, London

## Abstract

**Background:**

Critically ill trauma patients with severe injuries are at high risk for venous thromboembolism (VTE) and bleeding simultaneously. Currently, the optimal VTE prophylaxis strategy is unknown for trauma patients with a contraindication to pharmacological prophylaxis because of a risk of bleeding.

**Methods and Findings:**

Using decision analysis, we estimated the cost effectiveness of three VTE prophylaxis strategies—pneumatic compression devices (PCDs) and expectant management alone, serial Doppler ultrasound (SDU) screening, and prophylactic insertion of a vena cava filter (VCF)—in trauma patients admitted to an intensive care unit (ICU) with severe injuries who were believed to have a contraindication to pharmacological prophylaxis for up to two weeks because of a risk of major bleeding. Data on the probability of deep vein thrombosis (DVT) and pulmonary embolism (PE), and on the effectiveness of the prophylactic strategies, were taken from observational and randomized controlled studies. The probabilities of in-hospital death, ICU and hospital discharge rates, and resource use were taken from a population-based cohort of trauma patients with severe injuries (injury severity scores >12) admitted to the ICU of a regional trauma centre. The incidence of DVT at 12 weeks was similar for the PCD (14.9%) and SDU (15.0%) strategies, but higher for the VCF (25.7%) strategy. Conversely, the incidence of PE at 12 weeks was highest in the PCD strategy (2.9%), followed by the SDU (1.5%) and VCF (0.3%) strategies. Expected mortality and quality-adjusted life years were nearly identical for all three management strategies. Expected health care costs at 12 weeks were Can$55,831 for the PCD strategy, Can$55,334 for the SDU screening strategy, and Can$57,377 for the VCF strategy, with similar trends noted over a lifetime analysis.

**Conclusions:**

The attributable mortality due to PE in trauma patients with severe injuries is low relative to other causes of mortality. Prophylactic placement of VCF in patients at high risk of VTE who cannot receive pharmacological prophylaxis is expensive and associated with an increased risk of DVT. Compared to the other strategies, SDU screening was associated with better clinical outcomes and lower costs.

*Please see later in the article for Editors' Summary*

## Abstract 2

Using decision analysis, Henry Stelfox and colleagues estimate the cost-effectiveness of three venous thromboembolism prophylaxis strategies in patients with severe traumatic injuries who were also at risk for bleeding complications.

## Editors' Summary

**Background:**

For patients who have been seriously injured in an accident or a violent attack (trauma patients), venous thromboembolism (VTE)—the formation of blood clots that limit the flow of blood through the veins—is a frequent and potentially fatal complication. The commonest form of VTE is deep vein thrombosis (DVT). “Distal” DVTs (clots that form in deep veins below the knee) affect about half of patients with severe trauma; “proximal” DVTs (clots that form above the knee) develop in one in five trauma patients. DVTs cause pain and swelling in the affected leg and can leave patients with a painful condition called post-thrombotic syndrome. Worse still, part of the clot can break off and travel to the lungs where it can cause a life-threatening pulmonary embolism (PE). Distal DVTs rarely embolize but, if untreated, half of patients who present with a proximal DVT will develop a PE, and 2%–3% of them will die as a result.

**Why Was This Study Done?:**

VTE is usually prevented by using heparin, a drug that stops blood clotting, but clinicians treating critically ill trauma patients have a dilemma. Many of these patients are at high risk of serious bleeding complications so cannot be given heparin to prevent VTE. Nonpharmacological ways to prevent VTE include the use of pneumatic compression devices to keep the blood moving in the legs (clots often form in patients confined to bed because of the sluggish blood flow in their legs), repeated screening for blood clots using Doppler ultrasound, and the insertion of a “vena cava filter” into the vein that takes blood from the legs to the heart. This last device catches blood clots before they reach the lungs but increases the risk of DVT. Unfortunately, no-one knows which VTE prevention strategy works best in trauma patients who cannot be given heparin. In this study, therefore, the researchers use decision analysis (the systematic evaluation of the most important factors affecting a decision) to estimate the costs and likely clinical outcomes of these strategies.

**What Did the Researchers Do and Find?:**

The researchers used cost and clinical data from patients admitted to a Canadian trauma center with severe head/neck and/or abdomen/pelvis injuries (patients with a high risk of bleeding complications likely to make heparin therapy dangerous for up to two weeks after the injury) to construct a Markov decision analysis model. They then fed published data on the chances of patients developing DVT or PE, and on the effectiveness of the three VTE prevention strategies, into the model to obtain estimates of the costs and clinical outcomes of the strategies at 12 weeks after the injury and over the patients' lifetime. The estimated incidence of DVT at 12 weeks was 15% for the pneumatic compression device and Doppler ultrasound strategies, but 25% for the vena cava filter strategy. By contrast, the estimated incidence of PE was 2.9% with the pneumatic compression device, 1.5% with Doppler ultrasound, but only 0.3% with the vena cava filter. The expected mortality with all three strategies was similar. Finally, the estimated health care costs per patient at 12 weeks were Can$55,334 and Can$55,831 for the Doppler ultrasound and pneumatic compression device strategies, respectively, but Can$57,377 for the vena cava filter strategy; similar trends were seen for lifetime health care costs.

**What Do These Findings Mean?:**

As with all mathematical models, these findings depend on the data fed into the model and on the assumptions included in it. For example, because data from one Canadian trauma unit were used to construct the model, these findings may not be generalizable. Nevertheless, these findings suggest that, although VTE is common among patients with severe injuries, PE is not a major cause of death among these patients. They also suggest that the use of vena cava filters for VTE prevention in patients who cannot receive heparin should not be routinely used because it is expensive and increases the risk of DVT. Finally, these results suggest that, compared with the other strategies, serial Doppler ultrasound is associated with better clinical outcomes and lower costs.

**Additional Information:**

Please access these Web sites via the online version of this summary at http://dx.doi.org/10.1371/journal.pmed.1000098.

- The US National Heart Lung and Blood Institute provides information (including an animation) on deep vein thrombosis and pulmonary embolism
- MedlinePlus provides links to more information about deep vein thrombosis and pulmonary embolism (in several languages)
- The UK National Health Service Choices Web site has information on deep vein thrombosis and on embolism (in English and Spanish)
- The Eastern Association for the Surgery of Trauma working group document Practice Management Guidelines for the Management of Venous Thromboembolism in Trauma Patients can be downloaded from the Internet

## Introduction

Venous thromboembolism (VTE), including deep vein thrombosis (DVT) and pulmonary embolism (PE), are common complications that are associated with high rates of morbidity and mortality in trauma patients recovering from severe injuries. The risk of developing VTE is dependent on various factors including patient age and the type and severity of injury [1],[2]. On average, trauma patients have a 58% risk of distal DVT and an 18% risk of proximal DVT [2]. If left untreated, half of those who present with a proximal DVT will develop a clinically important PE, and of those, 2%–3% will die as a consequence of the PE [3],[4].

Prevention of VTE in trauma patients, who are at high risk for thrombosis and bleeding simultaneously, poses a major challenge [1]. As many as 22% of trauma patients have ongoing bleeding or injuries and are at high risk for serious bleeding complications [1],[5]. Furthermore, up to 86% of multiple trauma patients sustain injuries to the lower extremities, which may preclude the effective use of pneumatic compression devices (PCDs) [1]. The Eastern Association for the Surgery of Trauma (EAST) recommends consideration of vena cava filter (VCF) insertion in patients without documented DVT or PE who are at high risk of VTE and who cannot receive pharmacological prophylaxis [1]. Other professional trauma associations, including the British Trauma Society, Trauma Association of Canada, and the Australasian Trauma Society, provide no formal VTE prophylaxis guidelines. Nevertheless, a retrospective review of VCF insertions at 21 North American trauma centres suggests that significant variation exists in the frequency that VCFs are used in different trauma centres, with VCFs inserted twice as frequently at low-volume trauma centres as at high-volume centres [6]. VCFs are known to be effective at preventing PE in patients with known DVT [7], but their effectiveness and safety as prophylactic therapy for prevention of PE in patients at risk for DVT has not been tested in a randomized controlled trial. This is an important concern given that the use of VCF increases the risk of DVT [8], and that DVTs may result in long-term complications, including severe venous stasis and ulceration [9],[10]. Currently, the optimal prophylaxis strategy for patients at high risk of VTE and a contraindication to pharmacological prophylaxis is unknown. Considering the estimated cost of VCF (Can$3,600 per insertion and removal), the potential frequency of its use [11], and the uncertainty regarding its effectiveness and safety [12],[13], decision-makers must determine its optimal use.

We collected clinical and cost information on a cohort of trauma patients admitted to an intensive care unit (ICU) with severe injuries who were believed to have a contraindication to pharmacological VTE prophylaxis for two weeks because of a risk of major bleeding. Based on this cohort of patients, using decision analysis, we estimated the cost effectiveness of three VTE prophylaxis strategies: PCDs and expectant management alone, serial Doppler ultrasound (SDU) screening, and prophylactic insertion of a VCF.

## Methods

### Study Design

Using decision analysis and an analytic horizon of a lifetime, we calculated the cost effectiveness of three different VTE prophylaxis strategies in trauma patients with severe injuries admitted to the ICU who were believed to have a contraindication to pharmacological VTE prophylaxis for up to 2 wk because of a risk of major bleeding. The three strategies considered were: (1) PCDs and expectant management alone; (2) SDU screening; and (3) prophylactic insertion of a VCF.

We modelled the analysis over the course of 30 y since the average age of our severely injured cohort was 40 y. Given the importance of short-term clinical outcomes in trauma patients, we also estimated the incidence of DVTs, PEs, deaths, and health care costs at 12 wk for the three strategies. In base case analyses, we took the perspective of the health care purchaser. Costs and quality-adjusted life years (QALYs) were discounted at 5% annually, and costs were inflated to 2007 Canadian dollars (Can$1.00 = US$0.96) using the Bank of Canada online inflation calculator.

### Cohort of Trauma Patients on Which the Decision Analysis Was Based

Guidelines published by EAST and the Brain Trauma Foundation suggest that trauma patients with intracranial haemorrhage, ocular injury with associated haemorrhage, solid intra-abdominal injury (i.e., liver, spleen, kidney), or pelvic or retroperitoneal hematoma requiring transfusion are at increased risk of bleeding complications for 5–10 d following injury [1],[14]. Patients who have ongoing bleeding, are at high risk of bleeding, or will not tolerate even minor bleeding have a contraindication to pharmacological VTE prophylaxis. Estimates of the risk of death and cost of care in these subgroups of trauma patients with severe injuries are unknown. Therefore, we identified a cohort of trauma patients admitted to ICU with severe head/neck and abdomen/pelvis injuries who, according to the EAST and Brain Trauma Foundation guidelines, would be at risk of serious bleeding complications and would be likely to have a contraindication to pharmacological VTE prophylaxis to obtain accurate estimates of mortality and direct health care costs. Therefore, we undertook a cohort study to obtain accurate estimates of mortality and direct health care costs for trauma patients admitted to ICU with severe head/neck and abdomen/pelvis injuries that, according to the EAST and Brain Trauma Foundation guidelines, would be at risk of serious bleeding complications and would be likely to have a contraindication to pharmacological VTE prophylaxis. The Conjoint Health Research Ethics Board at the University of Calgary and Calgary Health Region approved this study.

Foothills Medical Centre is the sole regional adult trauma referral centre in Southern Alberta (population 1.5 million) and admits over 1,000 patients each year with severe injuries (injury severity score>12) [15]. We based our analysis on a cohort of adult (age≥15 y) patients admitted to the trauma centre's multisystem ICU between 01 April 2001 and 28 March 2006 with an admitting diagnosis of traumatic injury who were not treated with prophylactic VCF insertion. Patients were included if their admitting diagnosis was a traumatic injury, their injury severity score (ISS) was greater than 12 and their head/neck or abdomen/pelvis abbreviated injury scale (AIS) score was 3 or greater [15]. We selected this cohort of trauma patients because their injuries most closely approximated those identified by the EAST and Brain Trauma Foundation guidelines as being at high risk of serious bleeding complications and a potential contraindication to pharmacological VTE prophylaxis (Table 1) [1],[14].

**Table 1:** Patient characteristics, costs, and outcomes.

Characteristic	Subcategory	All Patients (*n* = 1,015)
**Age, y**		39.3 (38.1–40.5)
**Male, ** ***n*** ** (%)**		775 (76)
**Mechanism of injury, ** ***n*** ** (%)**	Motor vehicle collision	610 (60)
	Fall	224 (22)
	Violence	106 (10)
	Other	76 (7)
**Glasgow coma score**	Scene	6.9 (6.7–7.1)
	Emergency department	6.2 (6.0–6.5)
**Injury severity score**		30.5 (29.8–31.2)
**Abbreviated injury severity score** a	Head/neck	4.3 (4.2–4.3)
	Face	2.0 (2.0–2.1)
	Chest	3.0 (2.9–3.1)
	Abdomen/pelvis	3.3 (3.2–3.4)
	Extremities	2.6 (2.6–2.7)
	External	1.1 (1.0–1.1)
**Ethanol screen positive, ** ***n*** ** (%)**		317 (31)
**APACHE II score**		20.7 (20.2–21.1)
**SOFA score** b		12.6 (12.3–12.9)
**TISS score**		29.7 (29.0–30.5)
**Surgery during hospital stay, ** ***n*** ** (%)**		342 (34)
**Duration of mechanical ventilation, median (interquartile range), d**		3 (1–9)
**Length of stay, median (interquartile range), d**	ICU	5 (2–11)
	Hospital	17 (7–44)
**Mortality, ** ***n*** ** (%)**	48 h from admission	122 (12)
	ICU discharge	232 (23)
	Hospital discharge	242 (24)
**Cost of care, median (interquartile range), Can$**	ICU	9,645 (4,097–25,966)
	Ward	23,378 (11,026–49,535)
	Total hospital stay	35,282 (18,196–74,168)
**Annual cost of care for hospital survivors (years 1–3), Can$**	Year 1	3,460 (2,139–4,780)
	Year 2	1,100 (453–1,746)
	Year 3	537 (169–905)

Data are presented as means (95% confidence intervals) unless otherwise indicated.

a Abbreviated injury severity scores are provided for patients with documented injuries involving the head/neck (*n* = 946), face (*n* = 335), chest (*n* = 528), abdomen/pelvis (*n* = 345), extremities (*n* = 417), and external (*n* = 238) body regions.

b SOFA (Sequential Organ Failure Assessment) score calculated at 07:00 on first day of admission to the ICU.

APACHE, Acute Physiology and Chronic Health Evaluation; TISS, Therapeutic Intervention Scoring System.

### Prophylaxis Strategies

Patients treated with PCDs received expectant management for VTE during the first 2 wk of hospital admission unless a DVT or PE was detected clinically and subsequently diagnosed radiographically. In the second strategy, all patients received PCDs as well as weekly SDU screening for the duration of their hospitalization beginning in the first week of ICU admission. Patients with a positive test (true positive or false positive) were assumed to have a DVT. In the final strategy, patients underwent prophylactic VCF insertion within 48 h of admission. Patients who died within 48 h of admission were assumed to have died before VCF insertion and from causes unrelated to VTE.

In all three strategies, based on recommendations from the EAST guidelines that high risk trauma patients were at risk of bleeding for 5–10 d following initial injury, contraindications to pharmacological VTE prophylaxis were assumed to resolve after 2 wk of hospitalization, and patients in each strategy then initiated prophylaxis with low-molecular weight heparin [1]. Patients diagnosed with a DVT in the PCD and SDU screening strategies were treated with VCF insertion during the first 2 wk of hospitalization and with therapeutic anticoagulation thereafter. In the VCF strategy patients were assumed to receive therapeutic anticoagulation during their hospital stay only if they developed a PE and not just a DVT, because the risk of a PE with a VCF is very low. All patients diagnosed with VTE after 2 wk were assumed to receive initial anticoagulation with low-molecular weight heparin and subsequent ongoing anticoagulation with warfarin for a 1-y period after hospital discharge.

### Markov Analysis

**Figure 1:**
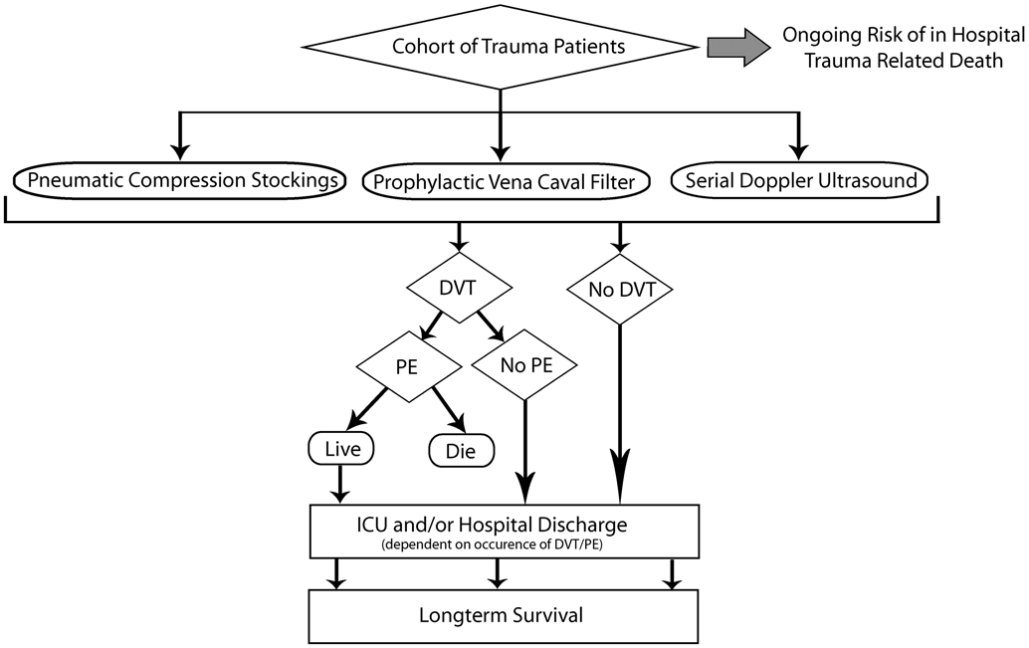
Model of three venous thromboembolism prophylaxis strategies.

Among the different types of decision analysis, the most appropriate method to model recurring events and transitions between different health states is Markov analysis [16]. Since our analysis modelled recurring weekly risks of DVT, death, and transition from the ICU to the hospital and subsequently home, we selected Markov analysis (Data Pro software, TreeAge software, Williamstown) (Figure 1). Through the use of multiple health states and decision analytic software, the probabilities of transitions between health states can be allowed to vary over the course of weekly Markov cycles [16]. In Markov analysis, a model is constructed, ideally using costs and clinical outcome data from an actual patient cohort (cohort of trauma patients in our study), which replicates these clinical outcomes and costs. Next, tests or treatments (prophylaxis strategies in our study) are overlaid on the model, and the subsequent impact on clinical outcomes and costs can be accurately estimated [16]. Each health state can also be assigned a utility (i.e., a measure of overall quality of life), and the cumulative utility spent in each state can be summed to calculate the QALYs of each strategy. The starting point of our model was the cohort of trauma patients with severe injuries who were believed to have up to a 2-wk contraindication to pharmacologic prophylaxis.

### Clinical Effects

#### Probability of DVT

The probability of proximal DVT was taken from an observational study [2] and a randomized trial [17] comparing low-dose heparin with low-molecular weight heparin as prophylaxis against VTE after major trauma. This probability, which was used for the non-VCF strategies, was taken to be 6.4% weekly for the initial 2 wk in patients receiving no pharmacological VTE prophylaxis, and 3.1% weekly thereafter (while receiving low-molecular weight heparin as prophylaxis) (Table 2) [2],[17]. In all strategies, we assumed that 50% of all DVTs were asymptomatic [18], and would only be diagnosed in the SDU strategy or after development of a clinical PE. Patients with an asymptomatic undiagnosed DVT were assumed not to receive therapeutic anticoagulation.

**Table 2:** Base case probabilities and ranges considered.

Variable	Subcategory	Base Case Estimate	Range (95% Confidence Interval)	Reference
**Incidence of proximal DVT**	No prophylaxis	18% at 21 d	14%–22%	[2]
	Prophylaxis with VCF alone	OR 1.87	1.10–3.20	[7] a
	Pharmacological prophylaxis alone	6% at 14 d	2%–10%	[17] a
**Incidence of PE in patients with proximal DVT**	No treatment	50%	±25%	[19]
	Treatment with VCF alone	1.1% at 12 d	0.1%–3.9%	[7] a
	Pharmacological treatment alone	4.8% at 12 d	2.2%–8.9%	[7] a
**Fatality rate from PE**		2.5% at 14 d	1.2%–4.6%	[4]
**Complication rates**	Risk of death from VCF insertion	0.12%	0.0%–0.3%	[22]
	Risk of mild to moderate PTS	23.7%	14%–21%	[10]
	Risk of severe PTS	7.0%	3%–6%	[10]
	Risk of major bleeding from therapeutic anticoagulation	3.9% at 12 d	1.7%–7.5%	[7] a
**Test characteristics**	Probability that a proximal DVT is symptomatic	50	±25%	[18]
	Sensitivity of SDU in asymptomatic patients with DVT	0.61	0.51–0.73	[1]
	Specificity of SDU in asymptomatic patients with DVT	0.97	0.95–0.99	[1]
**Utility (a measure of overall quality of life, range 0–1)**	No VTE	0.691	—	[23]
	VTE with severe PTS	0.641	—	[9],[23]
**Annual discount rate**	Costs	5%	0%–6%	[39]
	Utilities	5%	0%–6%	[39]

a Randomised controlled trial.

Abbreviations: PTS, post-thrombotic syndrome.

#### Probability of PE

The probability of a patient with an undiagnosed DVT developing a PE was estimated to be 50% [19]. In patients with a known DVT who were treated with therapeutic anticoagulation the risk of developing a PE was documented as 4.8%, taken from a randomized trial [7]. Patients developing a PE had a subsequent early risk of PE-related death of 2.5% (Table 2) [4].

#### Clinical effects of VCF insertion

A systematic review of the literature was performed to determine the key probabilities of developing a proximal DVT, PE, or adverse event after prophylactic insertion of a VCF (see Text S1 for methods and full results, also Figure S1; Tables S1 and S2). A single randomized control trial was selected to derive probabilities because of its methodological rigor. In patients who had insertion of a VCF, the probability for development of in-hospital DVT were estimated to be 1.87-fold higher than the incidence of DVT in the non-VCF strategies, while the risk of developing a PE if a DVT developed was documented as 1.1% (Table 2) [7].

#### Mortality

The ICU and hospital weekly risk of death was estimated from the cohort study (Table 1). The risk of death for patients with DVTs was assumed to be higher only among those who developed a PE [20],[21]. Based on the results of our systematic review, we estimated a small (0.12%) risk of VCF insertion-related mortality [22]. After hospital discharge, the risk of death over 2 y was taken from an observational study documenting the survival of critically ill trauma patients discharged from hospital [23]. After 2 y, we assumed that survivors would return to the baseline risk of age-adjusted mortality for Canadians. We assumed similar mortality rates for patients discharged from hospital with VTE, compared to those with no VTE [20],[21].

#### Length of ICU and hospital stay

Within our cohort study, consistent with other published reports, length of stay was longer for patients who developed DVT and PE, although it is unclear whether VTE causes increased lengths of stay. Therefore, using content experts and a Delphi method, we estimated that ICU patients developing DVT or PE, and hospital patients developing DVT or PE, would have their discharge delayed by 2, 3, 5, and 7 d, respectively [24]. We assumed that the length of stay would not be increased for patients with a VCF who developed a DVT, given that they are at lower risk of PE, an assumption favourable to the prophylactic VCF strategy.

#### Other clinical effects

For diagnostic screening of proximal DVT with SDU, the sensitivity and specificity were derived from data published by EAST and reported to be 61% and 97% respectively in patients with serious traumatic injuries (Table 2) [1]. Utility estimates for long-term survivors of trauma were estimated using observational studies and were assumed to be lower for patients that developed severe post-thrombotic syndrome [9],[23].

### Health Care Costs

We estimated the cost of VCF insertion and SDU testing (both including physician fees) to be Can$2,310 and Can$386, respectively (Table 3). The cost of prophylactic low-molecular weight heparin and therapeutic anticoagulation was based on the Alberta Drug Benefit List (Table 3). We assumed that all patients receiving therapeutic anticoagulation would be at risk of major bleeding (3.9%) [7], and estimated the cost of managing this complication on a study from Heyland et al. [25]. Weekly ICU and trauma ward costs were obtained from the cohort of trauma patients, consistent with prior studies (Table 1) [26]. Mean weekly physician billings for these patients in ICU (Can$3,055) and on the Ward (Can$280) were obtained from previous estimates for a similar patient cohort (Table 3) [26]. The cost of non-VTE-related hospital readmissions for patients who survived to hospital discharge were estimated for the cohort of trauma patients. The cost of both PE and DVT related readmissions was taken from Aujesky et al. [27]. Outpatient VTE management costs were calculated on the basis of all relevant nursing, medication, lab, support staff, and supply-related costs, while the cost of managing patients with mild or severe venous ulcers for the 2 y following hospital discharge was estimated from a focused literature search (Tables 2 and 3).

**Table 3:** Base case patient costing estimates.

Variable	Subcategory	Cost (CAN $)	Range Tested	Source/Reference
**Inpatient Costs**				
**Prophylaxis**	VCF insertion (includes cost of two staff)	2,113	−50% to +100%	CHR microcosting [40]
	VCF removal	1,300		CHR microcosting [40]
	DU (bilateral)	240		CHR microcosting [40]
	Heparin (prophylactic dosage)/wk	308		ADB List [41]
**Physician fees (per patient)**	Mean weekly physician billings for patients (sepsis) in the ICU	3,055	±50%	[42]
	Mean weekly physician billings for patients (sepsis) on the ward	280	±50%	[42]
	Radiologist fee for DU scan	146		AMA fee schedule [43]
	Radiologist fee for insertion of VCF	197		AMA fee schedule [43]
**Cost of complications**	Major gastrointestinal bleed	9,195		[25]
**Cost of early VTE-related readmissions**	DVT	4,413		[27]
	PE	7,801		[27]
**Post-discharge Health Care Costs**				
**Monthly cost of outpatient anticoagulation**		159		ADB List [9],[41]
**Annual cost of severe PT**	Year 1	6,729		[9],[10]
	Year 2	2,956		[9],[10]
**Annual cost of mild/moderate PTS**	Year 1	1,479		[9],[10]
	Year 2	601		[9],[10]

Abbreviations: VCF, vena cava filter; CHR, Calgary Health Region; DU, Doppler ultrasound; ICU, intensive care unit; VTE, venous thromboembolism; DVT, deep venous thrombosis; PE, pulmonary embolism; PTS, post-thrombotic syndrome; AMA, Alberta Medical Association; ADB, Alberta Drug Benefit.

### Sensitivity Analysis

We subjected each of our estimates to rigorous sensitivity analysis using the ranges reported in Tables 2 and 3. In particular, we were interested in the impact of variations within the following variables: risk of DVT, the risk of a PE for patients with a DVT, and the risk of mortality associated with a PE. We explored two alternative estimates for the risk of developing a proximal DVT after prophylactic VCF insertion from studies whose patient populations closely matched our study cohort, but whose methodologies were case series [8],[28]. In our base case analysis, we did not account for removal of VCF, which at our facility (Foothills Medical Centre) costs Can$1,300 (Table 3). In sensitivity analysis, we estimated the impact of removing VCF from all patients who had no VTE at the time of hospital discharge.

## Results

The baseline characteristics, short-term clinical outcomes and associated costs of care for the cohort of trauma patients are shown in Table 1. The mean age of the patients was 39.3 y and the majority were male (76%). Motor vehicle collisions (60%) were the most common mechanism of injury followed by falls (22%) and violence (10%). The mean injury severity score of patients was 30.5, and the mean abbreviated injury severity scores for the head/neck and abdomen/pelvis were 4.3 and 3.3 respectively. One-third of the patients received surgery during their initial hospital stay. A total of 242 patients (24%) died before hospital discharge.

The results of the base case analysis are shown in Table 4. The incidence of DVT at 12 wk was similar for the PCD (14.9%) and SDU (15.0%) strategies, but was higher for the VCF (25.7%) strategy. Conversely, the incidence of PE was highest in the PCD strategy (2.9%), followed by the SDU (1.5%) and VCF (0.3%) strategies. Mortality at 12 wk was similar for all three strategies. All patients in the VCF strategy received a prophylactic VCF, while a minority of patients in the PCD (5.5%) and SDU (11.5%) strategies had VCFs inserted following the diagnosis of a DVT. Of note, in one-third of the patients in the SDU strategy (4.2% of total SDU cohort) with a VCF, it had been inserted following a false positive test. Health care costs at 12 wk were Can$55,831 for the PCD strategy, Can$55,334 for the SDU screening strategy, and Can$57,377 for the VCF strategy. Over a lifetime analysis, expected QALYs were similar for all three treatment strategies, although costs remained highest for the VCF strategy. In the base case analysis, the SDU screening strategy was dominant over the other strategies, as it was associated with better clinical outcomes and lower costs (both at 12 wk and over a lifetime time horizon).

**Table 4:** Clinical outcomes and costs for patients receiving three venous thromboembolism prophylaxis strategies.

Outcome	Subcategory	PCD	SDU	VCF
**Outcomes at 12 wk**	DVT, %	14.9	15.0	25.7
	PE, %	2.9	1.5	0.3
	Mortality, %	24.5	24.4	24.5
	VCF insertion, %	5.5	11.5	100
	Cost of ICU, hospital and subsequent care, Can$	55,831	55,334	57,377
**Outcomes over patient lifetime**	Cost of ICU, hospital and subsequent care, Can$	66,900	65,800	68,700
	Expected QALYs	6.9	6.9	6.9

Our analysis was not sensitive to plausible variations in the risk of developing a proximal DVT after prophylactic insertion of a VCF or in estimates of DVT and PE incidence (Table 5). It was also not sensitive to plausible variation in the risk of death associated with PE, or other variables. Moreover, the length of time that pharmacological VTE prophylaxis was contraindicated (2 or 4 wk) did not influence the results. In virtually all scenarios, the SDU strategy was optimal, in that it resulted in the lowest costs and best clinical outcomes. Removal of the VCF upon discharge from hospital added Can$637 to the VCF strategy.

**Table 5:** Sensitivity analysis of venous thromboembolism prophylaxis strategies.

Outcome^a^	PCDs	SDU	VCF
**Base case**			
Deep vein thrombosis, %	14.9	15.0	25.7
Pulmonary embolism, %	2.9	1.5	0.3
Mortality, %	24.5	24.4	24.5
VCF insertion, %	5.5	11.5	100
Cost of ICU, hospital and subsequent care, Can$	55,831	55,334	57,377
**Low estimate risk of DVT in patients with VCF ** [**28**]			
Deep vein thrombosis, %	14.9	14.6	14.6
Pulmonary embolism, %	2.9	1.5	0.2
Mortality, %	24.5	24.4	24.5
VCF insertion, %	5.5	11.5	100
Cost of ICU, hospital and subsequent care, Can$	55,831	55,294	56,964
**High estimate risk of DVT in patients with VCF ** [**8**]			
Deep vein thrombosis, %	14.9	14.8	39.0
Pulmonary embolism, %	2.9	1.5	0.5
Mortality, %	24.5	24.4	24.6
VCF insertion, %	5.5	11.5	100
Cost of ICU, hospital and subsequent care, Can$	55,831	55,313	58,428
**Base case risk of DVT reduced by 25% in all strategies**			
Deep vein thrombosis, %	11.5	11.5	20.1
Pulmonary embolism, %	2.2	1.2	0.2
Mortality, %	24.4	24.4	24.5
VCF insertion, %	4.1	9.8	100
Cost of ICU, hospital and subsequent care, Can$	55,523	55,509	57,166
**Base case risk of DVT increased by 25% in all strategies**			
Deep vein thrombosis, %	18.2	18.3	30.9
Pulmonary embolism, %	3.6	1.9	0.3
Mortality, %	24.5	24.5	24.5
VCF insertion, %	6.9	13.1	100
Cost of ICU, hospital and subsequent care, Can$	56,127	55,187	57,582
**Base case risk of PE reduced by 50% in all strategies**			
Deep vein thrombosis, %	14.9	15.0	25.7
Pulmonary embolism, %	1.6	0.8	0.1
Mortality, %	24.4	24.4	24.5
VCF insertion, %	5.2	11.4	100
Cost of ICU, hospital and subsequent care, Can$	55,855	55,072	57,384
**Base case risk of PE increased by 50% in all strategies**			
Deep vein thrombosis, %	14.9	15.0	25.7
Pulmonary embolism, %	4.0	2.2	0.4
Mortality, %	24.5	24.5	24.5
VCF insertion, %	5.9	11.6	100
Cost of ICU, hospital and subsequent care, Can$	55,814	55,577	57,370
**Base case risk of death from PE reduced to 1% in all strategies** b			
Deep vein thrombosis, %	14.9	15.0	25.7
Pulmonary embolism, %	2.9	1.5	0.3
Mortality, %	24.4	24.4	24.5
VCF insertion, %	5.5	11.5	100
Cost of ICU, hospital and subsequent care, Can$	55,850	55,347	57,380
**Base case risk of death from PE increased to 10% in all strategies** b			
Deep vein thrombosis, %	14.9	15.0	25.7
Pulmonary embolism, %	2.9	1.5	0.3
Mortality, %	24.8	24.6	24.7
VCF insertion, %	5.5	11.4	100
Cost of ICU, hospital and subsequent care, Can$	55,737	55,271	57,362
**Pharmacological prophylaxis contraindication increased to 4 wk duration in all strategies**			
Deep vein thrombosis, %	17.1	17.6	29.0
Pulmonary embolism, %	3.3	1.6	0.3
Mortality, %	24.5	24.5	24.5
VCF insertion, %	9.1	18.3	100
Cost of ICU, hospital and subsequent care, Can$	55,996	56,562	57,369
**VCF removal upon discharge from hospital** c			
Deep vein thrombosis, %	14.9	15.0	25.7
Pulmonary embolism, %	2.9	1.5	0.3
Mortality, %	24.5	24.4	24.5
VCF insertion, %	5.5	11.5	100
Cost of ICU, hospital and subsequent care, Can$	55,831	55,334	58,014

Expected QALYs were 6.9 for all three strategies for all sensitivity analyses.

a All outcomes are reported at 12 wk.

b Base-line risk of death from PE in model is 2.5%.

c VCF removed from patients discharged home from hospital with no VTE in VCF strategy alone at cost $1,300.

## Discussion

Using decision analysis, we estimated the cost effectiveness of three VTE prophylaxis strategies; PCD and expectant management alone, weekly SDU screening and prophylactic insertion of a VCF in all trauma patients with severe injuries and a contraindication to pharmacological VTE prophylaxis for 2 wk. The results demonstrate that prophylactic insertion of a VCF results in a markedly higher incidence of DVTs and moderately lower incidence of PEs. Patient mortality was not substantially influenced by the VTE prophylaxis strategy. Weekly screening with SDU was the optimal strategy, resulting in the best clinical outcomes and lowest costs.

Our study provides further insight into the clinical implications of VTE prevention strategies in trauma patients with severe injuries who are at simultaneously high risk for thrombosis and bleeding. First, critically ill trauma patients are unlikely to benefit from routine prophylactic insertion of a VCF because the attributable risk of death from PE is small and offset by the potential risks of VCF insertion. A previous economic evaluation by Brasel et al. [29] concluded that both ultrasound screening and insertion of a VCF are effective at preventing PE in high-risk ICU patients. However, their study assumed that patients were concurrently managed with pharmacological VTE prophylaxis and that the risk of death from a treated PE was 8% and 32% for an untreated PE. Although, many studies in the literature quote the risk of death in patients with PE as being as high as 30% [1], the risk of death is often reported over the subsequent year and the cause of death in the vast majority of these patients is the underlying condition predisposing to the PE (malignancy, infection, cardiac disease) [4], rather than the PE itself. Second, the optimal VTE prophylaxis strategy in critically ill trauma patients with a contraindication to pharmacological VTE prophylaxis is early diagnosis of DVT. Nevertheless, screening SDU is limited by the operating characteristics of the test in asymptomatic trauma patients with a small number of patients receiving false positive tests and a larger number of patients false negative tests. Third, clinicians caring for trauma patients with severe injuries need to re-evaluate on a daily basis the risk tradeoff of bleeding and thrombosis in their high-risk patients. Currently the best scientific evidence for VTE prophylaxis is for pharmacological prophylaxis, yet there is very little evidence to guide clinicians about when it is safe to initiate pharmacological VTE prophylaxis in trauma patients with injuries characterized by high risk for serious bleeding complications [1],[14]. In fact, current evidence suggests that considerable practice variation exists as to the timing of initiation of pharmacological VTE prophylaxis in trauma patients with severe injuries and that randomized controlled studies are urgently needed to answer this important question [14],[30]. Fourth, consideration should be given to revising VTE prophylaxis protocols to reflect the likely boundaries of therapeutic strategies pending data from definitive studies. A review of the literature suggests that prophylactic insertion of a VCF is included in the standard VTE prophylaxis protocols of many trauma centres [31] and that utilization has increased over time [11],[32],[33],[34]. Amending VTE prophylaxis protocols to exclude prophylactic insertion of a VCF while emphasizing DVT screening and early initiation of pharmacological prophylaxis is likely to reduce the incidence of DVT and improve patient outcomes.

Those unfamiliar with decision analysis may find the modelling and statistical techniques to be complex and non-transparent. It should be noted that the most important sources of data for our model (risk of DVT, risk of PE) come from high-quality randomized trials. Moreover, using these high-quality estimates, a simple calculation can approximate the results of our Markov analysis. The attributable risk of death from PE in the PCD and VCF strategies are very small and can be estimated as 0.22% (18% risk of DVT×50% risk of PE in a patient who develops DVT×2.5% risk of death in a patient who develops PE) and 0.13% (29% risk of DVT×1.1% risk of PE in a patient who develops DVT×2.5% risk of death in a patient who develops PE+0.12% risk of death from VCF insertion), respectively. On the cost side, there is an immediate cost of Can$2,300 for VCF insertion and Can$1,300 for removal. The results are similar to those reported in Table 4 with differences primarily related to the clinical diagnosis of DVT in the PCD strategy and treatment with VCF or anticoagulation. Compared to SDU, prophylaxis of 100 patients with VCF would be expected to yield an additional 11 proximal DVTs, but prevent one PE at an extra cost of Can$204,300 (100×Can$57,377−Can$55,334) over 12 wk.

The results of our study need to be interpreted within the context of its limitations. First, our results are based on an economic analysis of patients admitted to a medical–surgical ICU at a regional trauma centre in Canada. It is unknown whether the results of an economic analysis performed in one centre or country can be generalized to others [35]. However, our cohort of patients had baseline characteristics and in-hospital mortality rates similar to those reported for trauma patients with severe injuries admitted to trauma centres in other developed countries [36],[37],[38]. Second, our results are based on a contraindication to pharmacological VTE prophylaxis that includes injuries and a timeframe that not all clinicians will agree with. Nevertheless, a less conservative definition of contraindications to pharmacological VTE prophylaxis allowing more patients to receive pharmacological prophylaxis earlier following injury would only make insertion of VCF look less efficacious and less cost effective. Despite these limitations, a strength of our analysis is that key variable estimates were derived from a systematic review of the literature and based on high-quality randomized controlled trials, and that the results were robust to plausible variation in the risk of all tested variables, suggesting that similar findings would be likely in other settings.

### Conclusion

Venous thromboembolism is a common complication in trauma patients with severe injuries. However, the attributable mortality due to PE in this patient population appears to be small. Prophylactic placement of VCF in patients at high risk of VTE who cannot receive pharmacological prophylaxis is expensive, is associated with an increased risk of DVT, and should not be routinely performed. Strategies employing screening with SDU appear more effective and less expensive.

## Supporting Information

Figure S1 **Article selection.**
(0.03 MB DOC)

Table S1 **Summary of methods of included studies.**
(0.10 MB DOC)

Table S2 **Summary of results of included studies.**
(0.10 MB DOC)

Text S1 **Summary of systematic review of vena cava filters for management of venous thromboembolism.**
(0.07 MB DOC)

## Abbreviations

DVT: deep venous thrombosis
EAST: Eastern Association for the Surgery of Trauma
ICU: intensive care unit
PCD: pneumatic compression device
PE: pulmonary embolism
QALY: quality-adjusted life year
SDU: serial Doppler ultrasound
VCF: vena cava filter
VTE: venous thromboembolism
