# Supplementary material for: An Economic Evaluation of Venous Thromboembolism Prophylaxis Strategies in Critically Ill Trauma Patients at Risk of Bleeding
Source: PLoS Med. 2009 Jun 23;6(6):e1000098. doi: 10.1371/journal.pmed.1000098 (PMC2695771; doi:10.1371/journal.pmed.1000098)
Supplement: Figure S1 — Article selection. (0.03 MB DOC) [file pmed.1000098.s001.doc]

Figure S1. Article Selection

565 unique articles

531 articles excluded based on screening of titles and abstracts

34 full text articles reviewed

29 articles excluded based on specific criteria

20 no control group
3 no outcomes reported
3 review articles

2 no relevant data
1 systematic review of a single previously identified RCT

3 duplicate publications

1 no control

.. Non-English language

15 articles satisfied inclusion criteria

250 duplicate articles excluded when databases merged

815 references identified
 446 Medline
 358 EMBASE
 8 EBM Reviews - Cochrane Central Register of Controlled Trials
 3 EBM Reviews - Cochrane Database of Systematic Reviews

0 EBM Reviews - Health Technology Assessment

446 Ovid MEDLINE® - 1950 to May Week 2 2008
358 EMBASE - 1980 to 2008 Week 21
8 EBM Reviews - Cochrane Central Register of Controlled Trials 2nd Quarter 2008
3 EBM Reviews - Cochrane Database of Systematic Reviews 1st Quarter 2008

0 EBM Reviews - Health Technology Assessment 2nd Quarter 2008

10 articles identified from the review of reference lists
