# Supplementary material for: An Economic Evaluation of Venous Thromboembolism Prophylaxis Strategies in Critically Ill Trauma Patients at Risk of Bleeding
Source: PLoS Med. 2009 Jun 23;6(6):e1000098. doi: 10.1371/journal.pmed.1000098 (PMC2695771; doi:10.1371/journal.pmed.1000098)
Supplement: Table S1 — Summary of methods of included studies. (0.10 MB DOC) [file pmed.1000098.s002.doc]

Table S1. Summary of methods of included studies

| **Source** | Setting | **Patients** | **N** | **Mean Age**  **(range)** | **%**  **Female** | **Study Design** | **Co-interventions** | **Screening strategies** |
| --- | --- | --- | --- | --- | --- | --- | --- | --- |
| Decousus  [1] | 44 centers,  France | Hospitalized with DVT & high risk of PE | 400 | 73 | 52 | Randomized control trial | Factorial randomization heparin vs. LMWH | Clinical suspicion |
| PREPIC  [2] | 44 centers,  France | Same cohort as  Decousus et al. | 400 | 73 | 52 | 8 year follow up of Decousus et al. RCT | 50% patients prescribed vitamin K antagonists | Telephone interviews |
| Velmahos  [3] | Trauma  centers | Trauma patients | 3,210 | NR | NR | Meta-analysis of 6 observational studies with controls | NR | NR |
| Webb  [4] | 1 hospital | Acetabular fracture & osteosynthesis | 51 | 33.5 (14-76) | 37 | Observational study, concurrent controls | LDH, low dose aspirin, compression stockings | Clinical suspicion |
| Rosner  [5] | 2 hospitals | Spinal reconstruction surgery | 161 | NR | NR | Observational study, historical controls | Compression stockings, PCD | Weekly DU |
| Obeid  [6] | 1 hospital | Bariatric surgery | 2,099 | 45 | 85 | Observational study, concurrent controls | Warfarin, PCD, enoxaparin | Clinical suspicion |
| Khansarinia  [7] | Level 1  trauma center | Patients in trauma registry with ISS >9 | 324 | 37.5 | 24 | Observational study, historical controls | LDH, PCD | Clinical suspicion |
| Gosin  [8] | Level 1  trauma center | Patients in trauma registry with ISS >15 | 598 | NR | NR | Observational study, historical & concurrent controls | NR | NR |
| Benevenia  [9] | 1 hospital | Lower extremity pathological fractures | 47 | 58 (20-79) | NR | Observational study, concurrent controls | Compression stockings, PCD | DU preop & prior to discharge |
| Aburahma  [10] | 1 hospital | Pregnant with new DVT | 26 | 24 (17-39) | 100 | Observational study, concurrent controls | Therapeutic dose heparin | DU>6 mo. post partum |
| Rogers  [11] | Level 1  trauma center | High impact lower extremity trauma | 940 | 39.6 | NR | Observational study,  concurrent controls | PCD | IP weekly; DU 30 d, 6 mos. & yearly on discharge |
| White  [12] | State of  California | Hospitalized for acute VTE | 74,319 | 63.2 | 54.9 | Observational study, concurrent controls | NR | NR |
| Rodriguez  [13] | 1 Hospital | Trauma patients at high risk of VTE | 120 | 42 | 35.4 | Observational study, historical controls | PCD, LDSH | Clinical suspicion |
| Gargiulo  [14] | 1 Hospital | BMI > 55 kg/m^2^ & open gastric bypass | 35 | NR | 83 | Observational study, concurrent controls | PCD compression stockings, LDH, | Clinical suspicion |
| Langan  [15] | Level 1  trauma center | Trauma registry | 3,788 | NR | NR | Observational study, concurrent controls | Subcutaneous heparin, PCD | Clinical suspicion |
| **Articles not Satisfying Inclusion Criteria, but Retained for Additional Analyses** | | | | | | | | |
| Rogers  [16]^†^ | Level 1  trauma center | Trauma patients | 132 | 39 | 27 | Case series | PCD | Same as Rogers et al., 1997[11] |
| Wojcik  [17]^†^ | Level 1  trauma center | Trauma patients | 64 | NR | NR | Case series | NR | Clinical suspicion |
| Becker  [18]^‡^ | Multiple  Hospitals | Mixed patients | 2,557 | NR | NR | Systematic review of 24 case series | 0-100% of studies | Described adequately in 2 out of 24 case series |

Abbreviations: DVT; deep vein thrombosis, PE; pulmonary embolism, LMWH; low molecular weight heparin, LDH; low dose heparin, PCD; pneumatic compression devices, DU; Doppler ultrasound, IP; impedance plethysmography, NR; none reported, NA; not applicable

†Articles retain for sensitivity analyses ‡Article retained for estimate of VCF complications

**References**

1. Decousus H, Leizorovicz A, Parent F, Page Y, Tardy B, et al. (1998) A clinical trial of vena caval filters in the prevention of pulmonary embolism in patients with proximal deep-vein thrombosis. Prevention du Risque d'Embolie Pulmonaire par Interruption Cave Study Group. N Engl J Med 338: 409-415.

2. Decousus H (2005) Eight-year follow-up of patients with permanent vena cava filters in the prevention of pulmonary embolism: the PREPIC (Prevention du Risque d'Embolie Pulmonaire par Interruption Cave) randomized study. Circulation 112: 416-422.

3. Velmahos GC, Kern J, Chan LS, Oder D, Murray JA, et al. (2000) Prevention of venous thromboembolism after injury: an evidence-based report--part II: analysis of risk factors and evaluation of the role of vena caval filters. J Trauma 49: 140-144.

4. Webb LX, Rush PT, Fuller SB, Meredith JW (1992) Greenfield filter prophylaxis of pulmonary embolism in patients undergoing surgery for acetabular fracture. J Orthop Trauma 6: 139-145.

5. Rosner MK, Kuklo TR, Tawk R, Moquin R, Ondra SL (2004) Prophylactic placement of an inferior vena cava filter in high-risk patients undergoing spinal reconstruction. Neurosurg Focus 17: E6.

6. Obeid FN, Bowling WM, Fike JS, Durant JA (2007) Efficacy of prophylactic inferior vena cava filter placement in bariatric surgery. Surg Obes Relat Dis 3: 606-608; discussion 609-610.

7. Khansarinia S, Dennis JW, Veldenz HC, Butcher JL, Hartland L (1995) Prophylactic Greenfield filter placement in selected high-risk trauma patients. J Vasc Surg 22: 231-235; discussion 235-236.

8. Gosin JS, Graham AM, Ciocca RG, Hammond JS (1997) Efficacy of prophylactic vena cava filters in high-risk trauma patients. Ann Vasc Surg 11: 100-105.

9. Benevenia J, Bibbo C, Patel DV, Grossman MG, Bahramipour PF, et al. (2004) Inferior vena cava filters prevent pulmonary emboli in patients with metastatic pathologic fractures of the lower extremity. Clin Orthop Relat Res: 87-91.

10. Aburahma AF, Boland JP (1999) Management of deep vein thrombosis of the lower extremity in pregnancy: a challenging dilemma. Am Surg 65: 164-167.

11. Rogers FB, Shackford SR, Ricci MA, Huber BM, Atkins T (1997) Prophylactic vena cava filter insertion in selected high-risk orthopaedic trauma patients. J Orthop Trauma 11: 267-272.

12. White RH, Zhou H, Kim J, Romano PS (2000) A population-based study of the effectiveness of inferior vena cava filter use among patients with venous thromboembolism. Arch Intern Med 160: 2033-2041.

13. Rodriguez JL, Lopez JM, Proctor MC, Conley JL, Gerndt SJ, et al. (1996) Early placement of prophylactic vena caval filters in injured patients at high risk for pulmonary embolism. J Trauma 40: 797-802; discussion 802-794.

14. Gargiulo NJ, 3rd, Veith FJ, Lipsitz EC, Suggs WD, Ohki T, et al. (2006) Experience with inferior vena cava filter placement in patients undergoing open gastric bypass procedures. J Vasc Surg 44: 1301-1305.

15. Langan EM, 3rd, Miller RS, Casey WJ, 3rd, Carsten CG, 3rd, Graham RM, et al. (1999) Prophylactic inferior vena cava filters in trauma patients at high risk: follow-up examination and risk/benefit assessment. J Vasc Surg 30: 484-488.

16. Rogers FB, Strindberg G, Shackford SR, Osler TM, Morris CS, et al. (1998) Five-year follow-up of prophylactic vena cava filters in high-risk trauma patients. Arch Surg 133: 406-411; discussion 412.

17. Wojcik R, Cipolle MD, Fearen I, Jaffe J, Newcomb J, et al. (2000) Long-term follow-up of trauma patients with a vena caval filter. J Trauma 49: 839-843.

18. Becker DM, Philbrick JT, Selby JB (1992) Inferior vena cava filters. Indications, safety, effectiveness. Arch Intern Med 152: 1985-1994.
