# Supplementary material for: An Economic Evaluation of Venous Thromboembolism Prophylaxis Strategies in Critically Ill Trauma Patients at Risk of Bleeding
Source: PLoS Med. 2009 Jun 23;6(6):e1000098. doi: 10.1371/journal.pmed.1000098 (PMC2695771; doi:10.1371/journal.pmed.1000098)
Supplement: Table S2 — Summary of results of included studies. (0.10 MB DOC) [file pmed.1000098.s003.doc]

Table S2. Summary of results of included studies

| **Source** | **Follow Up**  **(range)** | **DVT** | | | **PE** | | **VCF Complications** | **DVT Odds Ratio (95% CI)** | **PE Odds Ratio (95% CI)** | |
| --- | --- | --- | --- | --- | --- | --- | --- | --- | --- | --- |
|  |  | VCF | | **Control** | VCF | **Control** |  |  |  |  |
| Decousus[1] | 2 yrs. | 20.8%* | | 11.6%* | 3.4%* | 6.3%* | Site thromboses (n=16) | 1.87 (1.10-3.2) | 0.50 (0.19-1.33) | |
| PREPIC[2] | 8 yrs.^β^ | 35.7%* | | 27.5%* | 6.2%* | 15.1%* | Post-thrombotic syndrome: VCF group (70.3%), non VCF group (69.7%) | 1.52^‡^  (1.02-2.27) | 0.37^‡^ (0.17-0.79) | |
| Velmahos[3] | NR | NR | | NR | 2/321 | 64/2,889 | NR | NA | 0.28  (0.00, 1.05) | |
| Webb[4] | Mean 18 mo. | 1/24 | | 1/27 | 0/24 | 2/27 | Post-thrombotic syndrome: VCF group (n=3), non-VCF group (n=1) | 1.13  (0.01-92.10) | NA | |
| Rosner[5] | Up to 18 mo. | NR | | NR | 0/39 | 16/122 | Post-thrombotic complications (n=2) | NA | NA | |
| Obeid[6] | NR^φ^ | 3/246 | | 12/1,847 | 2/246 | 11/1,847 | NR | 1.89 (0.34- 7.06) | 1.37 (0.15-6.32) | |
| Khansarinia[7] | NR | NR | | NR | 0/108 | 13/216 | Vein thrombosis (n=1), migration (n=1) | NA | NA | |
| Gosin[8] | NR | NR | | NR | 0/99 | 16/499 | No complications | NA | NA | |
| Benevenia[9] | Mean 11.5 mo. (3-24 mo) | 2/24 | | 1/23 | 0/24 | 5/23 | Malposition (n=1), VCF occlusion (n=1), site thrombosis (n=1), haematoma (n=1) | 2.00 (0.10-123.26) | NA | |
| Aburahma[10] | Mean 61 mo. (18-102 mo.) | 0/11 | | 0/15 | 0/11 | 2/15 | Postthrombotic syndrome: VCF group (n=3), control group (n=4) | NA | NA | |
| Rogers[11] | NR | 3/35 | | NR | 1/35 | 1/905 | Insertion site thrombosis (n=2), VCF site thrombosis (n=1), VCF tilting (n=1) | NA | 26.59 (0.33-2,088.94) | |
| White[12] | 1 yr. | 8.7%^†^ | | 6.0%^†^ | 3.3%^†^ | 1.6%^†^ | NR | 1.14^‡^ (0.92-1.43) | 1.66^‡^  (0.91-3.06) | |
| Rodriguez[13] | NR | 6/40 | | 15/80 | 1/40 | 14/80 | No complications | 0.76 (0.22-2.33) | 0.12 (0.00-0.86) | |
| Gargiulo[14] | Up to 30 days post-op. | NR | | NR | 0/18 | 5/18 | NR | NA | NA | |
| Langan[15] | NR | 12.8% | | 1.6% | 0.5% | 0.13% | Improper placement (n=1), arterial fistula (n=1), haematoma (n=1) | 9.03 | 3.86 | |
| **Articles not Satisfying Inclusion Criteria, but Retained for Additional Analyses** | | | | | | | | | | |
| Rogers[16] | Length of stay | 12% | NA | | 2.3% | NA | Site thrombosis (n=4), filter tilt (n=7), strut malposition (n=50) | NA | | NA |
| Wojcik[17] | Mean 7 days | 44% | NA | | 0% | NA | Lower extremity swelling (n=3) | NA | | NA |
| Becker[18] | 1-11 mo. | NR | NR | | NR | NR | Risk of death from VCF insertion 0.12% | NA | | NA |

Abbreviations: DVT; deep vein thrombosis, PE; pulmonary embolism, NR; none reported, NA; not applicable, † Patients with no previous hospitalization for VTE

‡ Hazard ratio
* Outcomes assessors blinded
** Lead author blinded to patient grouping and clinical course
 7% attrition at 12 days
β Missing data on 4 patients
φ 6 patients presumed lost to follow-up

**References**

1. Decousus H, Leizorovicz A, Parent F, Page Y, Tardy B, et al. (1998) A clinical trial of vena caval filters in the prevention of pulmonary embolism in patients with proximal deep-vein thrombosis. Prevention du Risque d'Embolie Pulmonaire par Interruption Cave Study Group. N Engl J Med 338: 409-415.

2. Decousus H (2005) Eight-year follow-up of patients with permanent vena cava filters in the prevention of pulmonary embolism: the PREPIC (Prevention du Risque d'Embolie Pulmonaire par Interruption Cave) randomized study. Circulation 112: 416-422.

3. Velmahos GC, Kern J, Chan LS, Oder D, Murray JA, et al. (2000) Prevention of venous thromboembolism after injury: an evidence-based report--part II: analysis of risk factors and evaluation of the role of vena caval filters. J Trauma 49: 140-144.

4. Webb LX, Rush PT, Fuller SB, Meredith JW (1992) Greenfield filter prophylaxis of pulmonary embolism in patients undergoing surgery for acetabular fracture. J Orthop Trauma 6: 139-145.

5. Rosner MK, Kuklo TR, Tawk R, Moquin R, Ondra SL (2004) Prophylactic placement of an inferior vena cava filter in high-risk patients undergoing spinal reconstruction. Neurosurg Focus 17: E6.

6. Obeid FN, Bowling WM, Fike JS, Durant JA (2007) Efficacy of prophylactic inferior vena cava filter placement in bariatric surgery. Surg Obes Relat Dis 3: 606-608; discussion 609-610.

7. Khansarinia S, Dennis JW, Veldenz HC, Butcher JL, Hartland L (1995) Prophylactic Greenfield filter placement in selected high-risk trauma patients. J Vasc Surg 22: 231-235; discussion 235-236.

8. Gosin JS, Graham AM, Ciocca RG, Hammond JS (1997) Efficacy of prophylactic vena cava filters in high-risk trauma patients. Ann Vasc Surg 11: 100-105.

9. Benevenia J, Bibbo C, Patel DV, Grossman MG, Bahramipour PF, et al. (2004) Inferior vena cava filters prevent pulmonary emboli in patients with metastatic pathologic fractures of the lower extremity. Clin Orthop Relat Res: 87-91.

10. Aburahma AF, Boland JP (1999) Management of deep vein thrombosis of the lower extremity in pregnancy: a challenging dilemma. Am Surg 65: 164-167.

11. Rogers FB, Shackford SR, Ricci MA, Huber BM, Atkins T (1997) Prophylactic vena cava filter insertion in selected high-risk orthopaedic trauma patients. J Orthop Trauma 11: 267-272.

12. White RH, Zhou H, Kim J, Romano PS (2000) A population-based study of the effectiveness of inferior vena cava filter use among patients with venous thromboembolism. Arch Intern Med 160: 2033-2041.

13. Rodriguez JL, Lopez JM, Proctor MC, Conley JL, Gerndt SJ, et al. (1996) Early placement of prophylactic vena caval filters in injured patients at high risk for pulmonary embolism. J Trauma 40: 797-802; discussion 802-794.

14. Gargiulo NJ, 3rd, Veith FJ, Lipsitz EC, Suggs WD, Ohki T, et al. (2006) Experience with inferior vena cava filter placement in patients undergoing open gastric bypass procedures. J Vasc Surg 44: 1301-1305.

15. Langan EM, 3rd, Miller RS, Casey WJ, 3rd, Carsten CG, 3rd, Graham RM, et al. (1999) Prophylactic inferior vena cava filters in trauma patients at high risk: follow-up examination and risk/benefit assessment. J Vasc Surg 30: 484-488.

16. Rogers FB, Strindberg G, Shackford SR, Osler TM, Morris CS, et al. (1998) Five-year follow-up of prophylactic vena cava filters in high-risk trauma patients. Arch Surg 133: 406-411; discussion 412.

17. Wojcik R, Cipolle MD, Fearen I, Jaffe J, Newcomb J, et al. (2000) Long-term follow-up of trauma patients with a vena caval filter. J Trauma 49: 839-843.

18. Becker DM, Philbrick JT, Selby JB (1992) Inferior vena cava filters. Indications, safety, effectiveness. Arch Intern Med 152: 1985-1994.
