# Supplementary material for: An Economic Evaluation of Venous Thromboembolism Prophylaxis Strategies in Critically Ill Trauma Patients at Risk of Bleeding
Source: PLoS Med. 2009 Jun 23;6(6):e1000098. doi: 10.1371/journal.pmed.1000098 (PMC2695771; doi:10.1371/journal.pmed.1000098)
Supplement: Text S1 — Summary of systematic review of vena cava filters for management of venous thromboembolism. (0.07 MB DOC) [file pmed.1000098.s004.doc]

Summary of Systematic Review of Vena Cava Filters for Management of Venous Thromboembolism

A systematic review of the literature was performed to determine the key probabilities of developing a proximal DVT, PE and adverse event after prophylactic VCF placement. We searched Medline (1950 to May Week 2, 2008), EMBASE (1980 to Week 21, 2008) and EBM Reviews (Cochrane Central Register of Controlled Trials, Cochrane Database of Systematic Reviews, Health Technology Assessment) to identify relevant studies. Searches were restricted to human and English language articles and used combinations of the following search terms: thrombosis, pulmonary embolism, anticoagulants, filter, postoperative complications, incidence and prevalence. Appropriate wildcards were used in the search in order to account for plurals and variations in spelling. Additional articles were identified through reference lists. Studies were considered for inclusion if they included both an intervention group (VCF insertion) and a control group and presented at least one of the following measures of incidence; DVT, PE, or VCF related adverse events.

The search yielded 815 citations, 446 from MEDLINE, 358 from EMBASE and 11 from EBM Reviews. Thirty-four full-text articles met initial inclusion criteria and were retrieved for assessment. Ten articles were selected from review of the reference lists of retrieved articles. We identified 15 articles that satisfied our inclusion criteria published between 1950 and 2008 (Figure S1); 12 observational studies with either historical or concurrent controls[1,2,3,4,5,6,7,8,9,10,11,12], 1 meta-analysis of 6 observational studies of VCF placement in trauma patients[13] and 1 randomized control trial of VCF placement in patients with DVT with follow up at 2 years[14] and 8 years[15]. Reasons for exclusion included no control group (n=20), no outcomes reported (n=3), review article (n=3), no relevant data (n=2), and systematic review of a single previously identified randomized control trial (n=1). The quality of most studies satisfying the inclusion criteria was low with control groups being non-equivalent to the intervention groups, co-interventions not reported, screening strategies for DVT and PE performed at the discretion of the clinicians, outcome assessors not blinded and patient follow up either not reported or reported solely for the duration of patients’ acute care hospitalization (Table S1). Data on the incidence of DVT, PE and VCF complications were abstracted from each article and reported as proportions (Table S2). The odds ratios for developing a DVT and PE were abstracted from each article, or calculated as crude odds ratios using the available data.

The single randomized control trial of VCF placement in patients with newly diagnosed DVTs was selected to derive probabilities because of its methodological rigor. In patients who had a VCF placed the base case probabilities for development of in-hospital DVT were estimated at 1.87 times the incidence of DVT in the non-VCF strategies while the risk of developing a PE if a DVT developed was documented as 1.1% at 12 days (3.4% at 2 years)[14]. Although failing to meet all inclusion criteria due to the lack of a control group, we identified two relevant case series which reported on the specific cohort of interest (trauma patients with contraindication to anticoagulation who received prophylactic VCF insertion); these reports were used to guide sensitivity analyses on the probability of developing a proximal DVT after prophylactic VCF placement[16,17]. A third article, a systematic review of 24 case series, was also included despite the lack of a control group as it provided the best estimate of the risk of death from VCF insertion[18].

**References**

1. Webb LX, Rush PT, Fuller SB, Meredith JW (1992) Greenfield filter prophylaxis of pulmonary embolism in patients undergoing surgery for acetabular fracture. J Orthop Trauma 6: 139-145.

2. Rosner MK, Kuklo TR, Tawk R, Moquin R, Ondra SL (2004) Prophylactic placement of an inferior vena cava filter in high-risk patients undergoing spinal reconstruction. Neurosurg Focus 17: E6.

3. Obeid FN, Bowling WM, Fike JS, Durant JA (2007) Efficacy of prophylactic inferior vena cava filter placement in bariatric surgery. Surg Obes Relat Dis 3: 606-608; discussion 609-610.

4. Khansarinia S, Dennis JW, Veldenz HC, Butcher JL, Hartland L (1995) Prophylactic Greenfield filter placement in selected high-risk trauma patients. J Vasc Surg 22: 231-235; discussion 235-236.

5. Gosin JS, Graham AM, Ciocca RG, Hammond JS (1997) Efficacy of prophylactic vena cava filters in high-risk trauma patients. Ann Vasc Surg 11: 100-105.

6. Benevenia J, Bibbo C, Patel DV, Grossman MG, Bahramipour PF, et al. (2004) Inferior vena cava filters prevent pulmonary emboli in patients with metastatic pathologic fractures of the lower extremity. Clin Orthop Relat Res: 87-91.

7. Aburahma AF, Boland JP (1999) Management of deep vein thrombosis of the lower extremity in pregnancy: a challenging dilemma. Am Surg 65: 164-167.

8. Rogers FB, Shackford SR, Ricci MA, Huber BM, Atkins T (1997) Prophylactic vena cava filter insertion in selected high-risk orthopaedic trauma patients. J Orthop Trauma 11: 267-272.

9. White RH, Zhou H, Kim J, Romano PS (2000) A population-based study of the effectiveness of inferior vena cava filter use among patients with venous thromboembolism. Arch Intern Med 160: 2033-2041.

10. Rodriguez JL, Lopez JM, Proctor MC, Conley JL, Gerndt SJ, et al. (1996) Early placement of prophylactic vena caval filters in injured patients at high risk for pulmonary embolism. J Trauma 40: 797-802; discussion 802-794.

11. Gargiulo NJ, 3rd, Veith FJ, Lipsitz EC, Suggs WD, Ohki T, et al. (2006) Experience with inferior vena cava filter placement in patients undergoing open gastric bypass procedures. J Vasc Surg 44: 1301-1305.

12. Langan EM, 3rd, Miller RS, Casey WJ, 3rd, Carsten CG, 3rd, Graham RM, et al. (1999) Prophylactic inferior vena cava filters in trauma patients at high risk: follow-up examination and risk/benefit assessment. J Vasc Surg 30: 484-488.

13. Velmahos GC, Kern J, Chan LS, Oder D, Murray JA, et al. (2000) Prevention of venous thromboembolism after injury: an evidence-based report--part II: analysis of risk factors and evaluation of the role of vena caval filters. J Trauma 49: 140-144.

14. Decousus H, Leizorovicz A, Parent F, Page Y, Tardy B, et al. (1998) A clinical trial of vena caval filters in the prevention of pulmonary embolism in patients with proximal deep-vein thrombosis. Prevention du Risque d'Embolie Pulmonaire par Interruption Cave Study Group. N Engl J Med 338: 409-415.

15. Decousus H (2005) Eight-year follow-up of patients with permanent vena cava filters in the prevention of pulmonary embolism: the PREPIC (Prevention du Risque d'Embolie Pulmonaire par Interruption Cave) randomized study. Circulation 112: 416-422.

16. Wojcik R, Cipolle MD, Fearen I, Jaffe J, Newcomb J, et al. (2000) Long-term follow-up of trauma patients with a vena caval filter. J Trauma 49: 839-843.

17. Rogers FB, Strindberg G, Shackford SR, Osler TM, Morris CS, et al. (1998) Five-year follow-up of prophylactic vena cava filters in high-risk trauma patients. Arch Surg 133: 406-411; discussion 412.

18. Becker DM, Philbrick JT, Selby JB (1992) Inferior vena cava filters. Indications, safety, effectiveness. Arch Intern Med 152: 1985-1994.
